# Inspecting the Retina: Oculomotor Patterns and Accuracy in Fundus Image Interpretation by Novice Versus Experienced Eye Care Practitioners

**DOI:** 10.3390/jemr19010011

**Published:** 2026-01-21

**Authors:** Suraj Upadhyaya

**Affiliations:** Chicago College of Optometry, Midwestern University, 555 31st Street, Downers Grove, IL 60515, USA; supadh@midwestern.edu; Tel.: +1-(630)-960-3026

**Keywords:** fundus image interpretation, retina, visual search, diagnostic accuracy, experienced, novices

## Abstract

Visual search behavior, influenced by expertise, prior knowledge, training, and visual fatigue, is crucial in ophthalmic diagnostics. This study investigates differences in eye-tracking strategies between novice and experienced eye care practitioners during fundus image interpretation. Forty-seven participants, including 37 novices (first- to fourth-year optometry students) and 10 experienced optometrists (≥2 years of experience), viewed 20 fundus images (10 normal, 10 abnormal) while their eye movements were recorded using an Eyelink1000 Plus gaze tracker (2000 Hz). Diagnostic and laterality accuracy were assessed, and statistical analyses were conducted using Sigma Plot 12.0. Results showed that experienced practitioners had significantly higher diagnostic accuracy (83 ± 6.3%) than novices (70 ± 12.9%, *p* < 0.005). Significant differences in oculomotor behavior were observed, including median latency (*p* < 0.001), while no significant differences were found in median peak velocity (*p* = 0.11) or laterality accuracy (*p* = 0.97). Diagnostic accuracy correlated with fixation count in novices (r = 0.54, *p* < 0.001), while laterality accuracy correlated with total dwelling time (r = −0.62, *p* < 0.005). The experienced practitioners demonstrated systematic and focused visual search patterns, whereas the novices exhibited unorganized scan paths. Enhancing training with visual feedback could improve fundus image analysis accuracy in novice clinicians.

## 1. Introduction

Images are extensively utilized in the healthcare industry, and efficient analysis of these images is crucial for diagnosis and disease management. The ability to detect retinal abnormalities is fundamental for eye care practitioners. Visual search behavior aids us in understanding object perception, and factors such as training and prior knowledge can influence this behavior, potentially affecting diagnosis and management outcomes.

Visual search behavior has shown promise in understanding human object perception across various scenarios. Analyzing the eye movement patterns of experienced practitioners may be beneficial in developing effective training models for clinicians with less experience [1]. Eye movement patterns can also be used to track the progress of training, as demonstrated by the data on difference in mean completion time for expert and novice surgeons in laparoscopic surgery [2]. This is particularly relevant for optometrists who utilize imaging modalities in their day to day practice. Developing an effective visual search strategy could enhance the accuracy of visual diagnosis and aid in reducing time for the management of ocular pathologies. Visual search behavior has been studied for a long time, back to Yarbus’ era [3].

The cognitive theory of visual expertise suggests that experts, through extensive experience, learn to identify and focus on key visual features that are specific to their field [4,5]. They develop mental schemas that allow them to process complex information more efficiently than novices, often relying on pattern recognition rather than detailed analysis. This framework, applicable across fields like radiology, sports, and art, helps explain not only what experts see but how they see it, revealing differences in cognitive and perceptual processing between experts and novices.

This processing is also different between different domains, in sports, arts, and medicine. Some focus more on accuracy and some on speed, which leads to different eye movement metrics to look into; for example, if the domain is sports, we look at the velocity of eye movements and latency of eye movements [6], and when it is medicine, we look into the total amount of time spent in the area of interest (AOI), focusing more on the efficient global–local processing of scenes [7,8].

Optometrists increasingly use digital fundus photography to diagnose and manage ocular diseases. Early detection of conditions like diabetic and hypertensive retinopathies is crucial. Accurate interpretation of fundus images is essential to reduce ocular disease burden and improve population health. Various studies have provided data suggesting that the signature of eye movements is present in experts’ oculomotor behavior and is more precisely detected by machine learning algorithms, whether it is while watching games or looking at art work [9,10]. In a study comparing novices and experts in interpreting maps, experts had a short fixation duration and a higher fixation rate, suggesting that they can interpret a larger part of visual scenes in the same time as a novice map interpreter [11]. Likewise, another study by Stein et al., investigating visual expertise while viewing sculptures among different levels of expertise, found mixed results in oculomotor dynamics between novices’ and experts’ eye movements [12]. It suggested that the differences in oculomotor behavior between naïve individuals and experts may be dependent on the professional domain [12].

Given these trends, education and expertise in retinal imaging remain critical components of oculomotor research. In the fields of ophthalmology and optometry, retinal images are essential for the diagnosis and management of ocular diseases. Additionally, when dealing with paired organs such as the eyes, knowing whether the diseased organ is the right or left eye is crucial [13]. This aspect is rarely mentioned in the medical literature, yet the consequences of such mistakes can be devastating [14]. Lawsuits due to operating on the wrong eye can lead to the loss of a clinician’s license and lifelong blindness for the patient.

Left and right discrimination is important in healthcare [15,16]. Laterality-based decisions are crucial in various medical procedures, such as surgery and diagnostic imaging, and still represent a significant source of medical errors [17]. We hypothesized that there are differences in eye movements between novices and experienced clinicians while performing diagnosis and laterality tasks. The purpose of this study was to compare diagnostic abilities and laterality awareness between novices and experienced eye doctors. We examined the visual search behavior of both experienced optometrists and novice optometry students and analyzed their oculomotor behavior at different training levels. Some of these data have appeared before, in conference abstract form [18].

## 2. Methods

This cross-sectional prospective study received approval from the Institutional Review Board of Midwestern University (IRB no: 21002) and was conducted at the Chicago College of Optometry in Downers Grove, IL, USA. Participants provided written consent, and all research adhered to the principles outlined in the Declaration of Helsinki. Novices and experienced doctors examined a collection of normal and abnormal retinal images, and both temporal and spatial components of eye movement sequences were analyzed to examine the detailed search behavior.

### 2.1. Participants

Participants were recruited from an accredited optometry program in the United States. Two groups were formed: a novice group consisting 37 novice clinicians (10 first-year, 10 second-year, 10 third-year, and 7 fourth-year students) and an experienced group comprising 10 clinicians with more than two years of experience (6.9 ± 2.8 years of experience).

### 2.2. Apparatus

Eye movements were recorded using the Eyelink 1000plus eye tracker (SR Research Ltd., Kanata, ON, Canada), a non-invasive infrared eye tracker with a sampling frequency of 2000 Hz. The eye tracker was desktop-mounted, and all saccadic targets and retinal images were displayed on a Dell monitor with a resolution of 2048 × 1152 pixels and a refresh rate of 60 Hz at 57 cm. A chin rest and headrest were utilized to minimize head movements.

### 2.3. Stimuli

The visually guided saccadic target was a 1-degree black dot appearing at the center of the screen, moving randomly in horizontal and vertical directions after a specified fixation duration. Each dot moved a set distance in each direction a predetermined number of times.

The fundus stimuli consisted of ten normal fundus images from a high-resolution fundus image database [19]. A diabetic retinopathy retinal image dataset was obtained from publicly available sources [20]. These images were devoid of personal identifiers. All 20 fundus images were standardized to a resolution of 1130 × 1130 pixels with a 24-bit color depth. Fundus features that were relevant to diabetic retinopathy and necessary landmarks for fundus evaluation were included. In each fundus photograph, anatomical landmarks such as the optic disk, macula, major blood vessels, and other retinal regions were predetermined. These areas of interest (AOIs) were used for analyzing fixation at specific locations.

### 2.4. Procedure

Upon obtaining consent, participants were instructed to sit comfortably in front of a computer, with their chin resting on a chin rest and their head supported by a headrest. A nine-point calibration and validation process was conducted to ensure the eye tracker accurately detected and recorded eye movements. Any error exceeding 0.50 degrees was not accepted, aiming to maximize the accuracy of the participant’s gaze.

**Figure 1:**
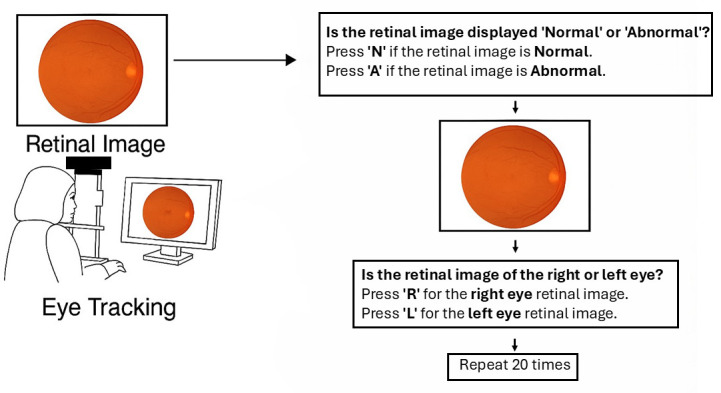
Procedural flow of the retinal image–viewing experiment. Participants first completed a saccadic eye-movement task, followed by the presentation and viewing of retinal images.

Figure 1 illustrates a user interface and workflow for retinal image classification using eye-tracking. On the left, a person is shown viewing a retinal image on a computer screen, with eye tracking technology monitoring their gaze. On the right, the image outlines a two-step decision process: first, the user determines whether the retinal image is “normal” or “abnormal” by pressing ‘N’ or ‘A’, respectively; second, they identify whether the image is of the right or left eye by pressing ‘R’ or ‘L’. This classification procedure is repeated 20 times with different retinal images. After completing the saccadic task, participants were recalibrated to ensure a stable gaze during the retinal image analysis phase. In the subsequent task, as illustrated in Figure 1, twenty retinal images were randomly presented, including ten normal (five right and five left fundus images) and ten abnormal (diabetic retinal images: five right and five left). Each retinal image was displayed for a maximum of 15 s, and participants were required to identify whether the image depicted a normal or abnormal retina by pressing “A” for abnormal or “N” for normal on the keyboard. Subsequently, the same retinal image was displayed for an additional 5 s, followed by a prompt asking participants to determine if the image represented the right or left eye by pressing either “R” or “L”. This process was repeated 20 times, with images randomly presented to all participants.

### 2.5. Data Processing and Analysis

Eye movement parameters, including saccade amplitude (in degrees), saccade peak velocity (in degrees/second), saccade latency (in milliseconds), fixation duration (in milliseconds), and fixation count (number), were collected as quantitative data. Saccadic latency is defined as the time delay between the appearance of a visual stimulus and the initiation of a saccadic eye movement. Fixation is defined as a period where the eye remains within a 1-degree visual angle for at least 100 milliseconds. Additionally, qualitative data such as scan path patterns were extracted from the eye tracker. The fixation rate was calculated using the ratio of dwelling time by fixation count on the fundus.

Qualitative data, such as scan path patterns, were subjectively evaluated. Diagnostic accuracy for normal and abnormal retinas, as well as laterality accuracy, which refers to the ability to correctly identify and differentiate between right or left retinal images, was recorded during the eye-tracking procedure for further analysis.

Statistical analyses were conducted to compare diagnostic accuracy and visual behavior between novice and experienced groups. Depending on data distribution, independent sample *t*-tests, Mann–Whitney U tests, or Mann–Whitney Rank Sum tests were used for group comparisons. One-way ANOVA and Kruskal–Wallis one-way ANOVA on ranks were applied for multi-group comparisons. Additionally, Pearson product–moment correlation coefficients were calculated to assess relationships between fixation metrics and diagnostic accuracy. All analyses were performed using SigmaPlot 12.0.

## 3. Results

A total of 47 participants took part in the study. The novice group consisted of 10 first-year optometry students (OD1), 10 second-year optometry students (OD2), 10 third-year optometry students (OD3), and 7 fourth-year optometry students (OD4), totaling 37 clinicians. Additionally, the experienced group comprised 10 experienced optometrists who were enrolled in the study.

### 3.1. Properties of Saccades and Fixation

Table 1 presents the mean latency and mean peak velocity of saccades with one standard deviation between the novice group and the experienced group. There was a significant difference in median latency between the novice group and the expert group (Mann–Whitney U test—T = 271,220.20, *p* < 0.001). However, there was no significant difference in the median peak velocity between the novice and experienced groups (Mann–Whitney Rank Sum test: U = 162,745.5, *p* = 0.11).

Analysis of oculomotor behavior across the novice groups (first-year to fourth-year students) revealed no significant differences in peak saccadic velocity. The Kruskal–Wallis one-way ANOVA on ranks yielded H(3) = 3.31, *p* = 0.34. Similarly, there was no significant difference in saccadic latency among the groups (H(3) = 2.46, *p* = 0.48), indicating comparable oculomotor control systems across all years.

### 3.2. Oculomotor Behavior

**Figure 2:**
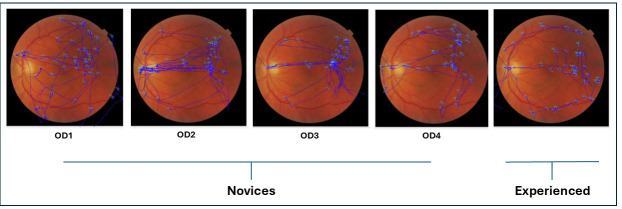
This figure depicts the overlay of raw eye movements executed by a representative novice and experienced participant while observing a left normal fundus image for 15 s before responding to a query regarding its normality or abnormality. The blue dots represent fixation points. The novice group comprises clinicians from year 1 to 4, designated as OD1, OD2, OD3, and OD4.

Figure 2 shows an overlay of raw eye movement data on the left fundus image from a representative participant in each group (novice and experienced). Each overlay illustrates the scan path recorded while the participant viewed the image for 15 s prior to indicating whether the fundus appeared normal or abnormal. Blue dots represent fixation points, and blue traces indicate the saccades connecting them.

### 3.3. Diagnostic Accuracy

**Figure 3:**
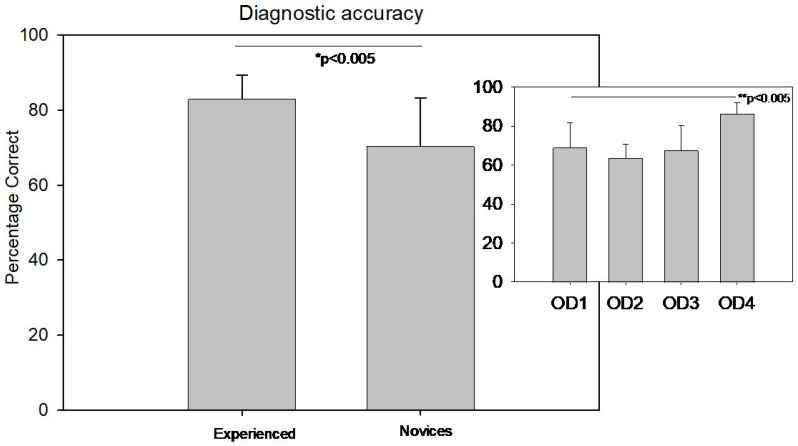
A vertical bar chart compares the average diagnostic accuracy percentages between experienced and novice observers, with their respective standard deviation. A small inset in the top right displays the distribution of diagnostic accuracy among different levels of clinicians from year 1 to 4. * and ** Shows statistical significance.

There was a significant difference in diagnostic accuracy between the experienced group (83 ± 6.3%) and the novice group (70 ± 12.9%) (Welch’s *t*-test T(30.7) = −4.3, *p* < 0.005, Cohen’s d= 2.25). Figure 3 displays the diagnostic accuracy comparison between the novice and experienced groups. The small inset in the top right of Figure 3 illustrates the distribution of diagnostic accuracy among different levels of clinicians from year 1 to 4.

In contrast, diagnostic accuracy showed a significant difference across the academic years. A one-way ANOVA revealed F(3,36) = 7.2, *p* < 0.001. Post hoc multiple pairwise comparisons using the Holm–Sidak method with the Bonferroni correction indicated that fourth-year students had significantly higher diagnostic accuracy than students in the first, second, and third years. No significant differences were observed between the first three years.

### 3.4. Laterality Accuracy

**Figure 4:**
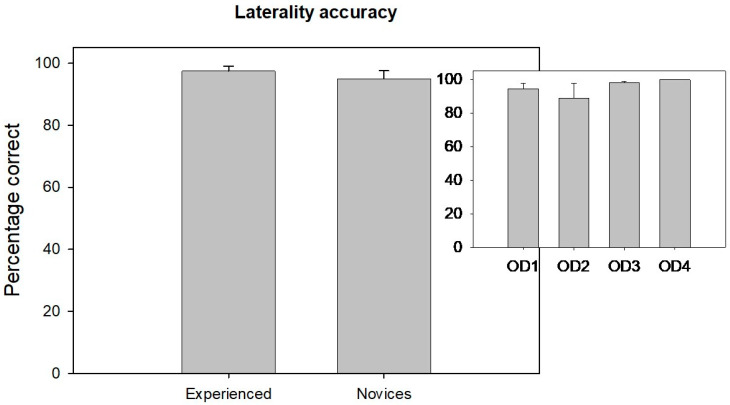
A vertical bar chart compares the average laterality accuracy percentages between experienced and novice observers, with their respective standard deviation. A small inset in the top right illustrates the distribution of laterality accuracy among different levels of novice clinicians from year 1 to 4.

Figure 4 displays the laterality accuracy comparison between the novice and experienced groups. There was no significant difference in laterality accuracy between the novice and experienced groups (Mann–Whitney Rank Sum test: U = 184, *p* = 0.97). The small inset in the top right side of Figure 4 illustrates the distribution of laterality accuracy among different levels of novice clinicians from years 1 to 4.

Laterality accuracy did not differ significantly across the groups (H(3) = 3.63, *p* = 0.30), suggesting that spatial localization skills are developed earlier and remain stable across training years.

### 3.5. Correlation Analysis

Table 2 shows the correlation matrix between fixation and diagnostic accuracy. There was no significant correlation between expert diagnostic accuracy and total dwelling time, total fixations, and fixation rate. However, the novices’ diagnostic accuracy showed significant correlations with total fixation count (Pearson product–moment correlation coefficient r = 0.54; *p* < 0.001). The Bonferroni correction is applied for multiple comparisons.

Table 3 shows the correlation matrix between fixation and laterality accuracy. There was no significant difference between the experienced laterality accuracy and total dwelling time, total fixation count, and total fixation rate. However, the novices’ laterality accuracy was significantly correlated with total dwelling time (Pearson product–moment correlation coefficient r = −0.62; *p* < 0.005). The Bonferroni correction is applied for multiple comparison.

### 3.6. Qualitative Analysis

**Figure 5:**
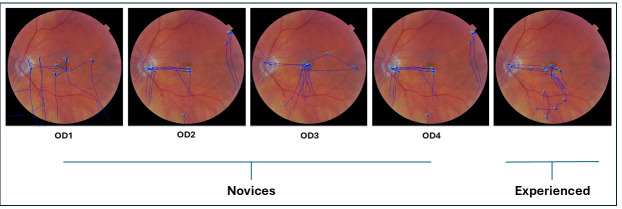
This figure depicts the overlay of raw eye movements executed by both the novice and experienced representative participants while viewing a left normal fundus image for 5 s before answering a question about whether the image is from the right or left fundus. All the blue dots represent fixation points. The novice group consists of clinicians from years 1 to 4, represented by OD1, OD2, OD3, and OD4.

The scan paths between novice participants and experienced participants differed during diagnostic accuracy assessments. Novice participants exhibited haphazard, unorganized, and non-goal-driven visual search behavior, as is evident in Figure 2 and Figure 5. In contrast, experienced participants demonstrated more organized and systematic visual search behavior, focusing on key structures such as the optic disk, macula, major blood vessels, and other retinal regions. Experienced participants took their time at each location, moving intentionally based on the question they were going to answer.

Figure 5 illustrates raw scan path patterns of novices and experienced participants while examining an abnormal retinal image for 5 s before answering questions about whether the fundus image belongs to the right or left eye. When examining the scan paths for laterality accuracy, as shown in Figure 5, both novice participants and experienced participants focused on the location of the optic nerve head and fovea to determine the laterality of the fundus images. However, the scan paths of the experienced participants were slightly more organized compared to those of the novice participants.

Table 4 shows the mean dwelling duration at each area of interest for the experienced and novice groups during the diagnostic accuracy experiment. During this experiment, both groups spent most of their time looking at other retinal regions. However, the experienced group spent significantly more time observing blood vessels compared to the novice group, while the novice group focused significantly more on the optic disk than the experienced group. There was no significant difference in the average amount of time both groups spent looking at the macula and other retinal regions.

Table 5 also presents the mean dwelling duration at each area of interest for the experienced and novice groups during the laterality accuracy experiment. In this experiment, the experienced group primarily focused on other retinal regions, whereas the novice group spent most of their time looking at the optic disk. There was no significant difference in the average amount of time both groups spent observing blood vessels and other retinal regions. However, the experienced group spent more time, on average, observing the macula, whereas the novice group focused significantly more on the optic disk than the experienced group.

## 4. Discussion

In this study, we explore the diagnostic and laterality accuracy differences between novices and experienced eye doctors while examining a series of normal and abnormal retinal images. Experienced participants demonstrated better diagnostic accuracy compared to novice participants. However, there was no significant difference in laterality accuracy between the two groups. Notably, significant differences in visual search behavior were observed between novice observers and experienced observers. Below, we will discuss some of these differences in the context of visual search behavior that was observed.

### 4.1. Influence of Oculomotor Behavior on Retinal Image Observation

The sequence of eye movements made while viewing an image, known as the scan path, reveals where saccades and fixations occur, providing insights into information gathering and helping to create a spatial fixation map [21,22]. Additionally, the scan path can unveil the cognitive processes underlying an individual’s examination of a scene [23]. A study by Stainer et al. examined the strategies used by experienced and novice clinicians to view the retina. They found that experienced clinicians used a holistic approach to process image information [24]. Another study demonstrated that a teaching intervention improved the gaze behavior of clinicians interpreting images of diabetic retinopathy [25]. O’Neill et al. showed that first-year residents had significantly longer total dwell times compared to third-year residents and specialists when looking for glaucomatous disks in the retina [26]. In our study, we observed a drastic improvement in the diagnostic accuracy of retinal images once clinicians reached their fourth year of training. Eye tracking, therefore, plays a crucial role in understanding the mechanisms of observation. Our findings suggest that as novices, clinicians gain more knowledge about retinal structures; their scan paths become more organized over the years of training, influencing the accuracy of their observations. The findings suggest that oculomotor control, as measured by the peak saccadic velocity and latency, remains consistent across the novice groups from first to fourth year. This implies that basic oculomotor functions are likely established early and do not significantly evolve with clinical training. However, diagnostic accuracy improves significantly with experience, as evidenced by the higher performance of fourth-year students. This supports the notion that clinical exposure and training enhance diagnostic capabilities over time. The lack of difference in diagnostic accuracy between first- and third-year students may reflect limited clinical experience during these early stages. The absence of significant differences in laterality accuracy across all years further supports the idea that spatial localization skills are acquired early and remain stable, independent of diagnostic training. Overall, while oculomotor control appears to be uniform across the novice groups, diagnostic accuracy is clearly influenced by clinical experience, highlighting the importance of progressive training in developing diagnostic competence.

Fixation patterns observed in eye-tracking studies offer valuable insight into the cognitive strategies underlying visual interpretation. Novices typically exhibit broader and more dispersed fixation behavior, reflecting an exploratory search driven by uncertainty and limited schema development. In contrast, experts demonstrate rapid, targeted fixations that reflect a shift from a slow, search-to-find strategy to a fast, holistic mode of perception. As shown by Kundel et al., this holistic strategy begins with a global analysis of the entire image, enabling proficient observers to identify diagnostically relevant perturbations—often within the first few seconds of viewing [27]. These early fixations are guided by a well-developed mental framework built through extensive experience and deliberate practice. Novices, lacking this perceptual foundation, rely more heavily on local feature analysis and exhaustive scanning, which leads to slower recognition and increased error rates. Thus, fixation behavior serves as a window into the reasoning processes that differentiate expert and novice performances, illustrating how perceptual efficiency evolves with expertise in medical image interpretation. Rubin’s study on radiologists’ gaze and accuracy in detecting lung nodules highlights the importance of understanding gaze patterns in all medical diagnostics [28]. Visual search errors, such as false negatives, can have significant consequences, particularly in medical diagnostics where missing signs of conditions like cancer can be costly. The societal costs associated with missed targets in various fields underscore the importance of improving visual search tasks. Providing observers with insights into their gaze patterns can help to address their shortcomings and improve their overall performance. Thus, improving the accuracy of diagnosis through training and technology is imperative.

The absence of a significant difference in laterality accuracy between novices and experts suggests that this task may rely more on foundational spatial reasoning than on advanced clinical experience. For example, identifying the orientation of the optic disk relative to the macula depends on consistent visual cues—such as the disk–macula axis and vessel arcades—that are emphasized early in optometric education and remain relatively uniform across fundus images. These characteristics enable even early-stage students to perform laterality judgments with reasonable accuracy. Consequently, this task may reach a performance ceiling quickly, limiting the observable improvement with experience and distinguishing it from more complex diagnostic tasks that require pattern recognition and clinical reasoning.

### 4.2. Implications for Training and Technology in Medical Education

Understanding and overcoming inattentional blindness could enhance students’ ability to analyze retinal images effectively. Additionally, eye-tracking technology has the potential to provide real-time feedback, improving visual search accuracy and efficiency. Poor visual scanning could lead to decision-making errors, which are often influenced by visual biases and allocation of attention [29]. We intentionally recruited future clinicians from various levels of training to investigate potential learning effects on oculomotor behavior as they gain more knowledge about retinal structures during their training. Indeed, our findings suggest that as the novice group learn more about retinal structure, their scan paths become more organized, indicating that knowledge of what to look for leads to more structured eye scanning. This is similar to Stofer and Che’s study, where novice performance improved with feedback and instructions while studying satellite data from the sea [30]. The accuracy of retinal image observation depends on the processes of searching, recognizing, and decision-making. Despite individuals often assuming they have thoroughly examined an image, they tend to shift their gaze to different parts of it. As seen in other studies, medical trainees with gaze training benefited more than the groups without gaze training [31]. In a similar study conducted by Shirley et al., although the percentage of missed areas of interest decreased after training, the accuracy in diagnosing diabetic retinal images did not change significantly [25]. We believe that this is due to their scoring system, which penalizes people for missing crucial signs of diabetic retinopathy in the retinal image, whereas our decision scores are correct percentages without any negative scoring for wrong judgment [25]. Eye-tracking technology can aid in assessing competency progression during medical education and training. By understanding and overcoming inattentional blindness, the novice group could improve their ability to analyze retinal images effectively. Guiding novice groups with feedback from skilled professionals can enhance their learning process and analysis skills.

The optic disk serves as a key landmark for determining the laterality of retinal images. In our laterality accuracy experiment, it was one of the two primary areas clinicians focused on to identify ocular laterality. Assessing both the optic disk and the macula is crucial for determining retinal image laterality. Automated systems frequently rely on the position of the optic disk to classify retinal images as belonging to the left or right eye, achieving high accuracy in laterality determination [32,33]. Additionally, combining the locations of the optic disk and macula is a common strategy in automated systems to enhance the accuracy of retinal image classification [34,35].

Our findings support the use of gaze data in developing adaptive training systems that enhance diagnostic education through AI and real-time feedback. By analyzing learners’ visual search behavior, these systems can compare scan paths to expert benchmarks and deliver personalized guidance—highlighting missed regions, inefficient patterns, or delayed attention to key anatomical features. Advances in eye-tracking hardware, machine learning, and real-time processing make this increasingly feasible. Early prototypes in radiology and surgical education by Brunyé et al. and Szulewski et al., respectively, demonstrate promising outcomes [36,37]. A robust framework could include expert gaze replays, real-time visual overlays, and adaptive feedback that prompts the review of overlooked areas like the macula. Training complexity can be dynamically adjusted based on gaze metrics and diagnostic accuracy, creating a personalized learning path. While not piloted in this study, our findings on novice–experienced clinicians’ differences in visual search behavior underscore the potential of such systems to improve fundus image interpretation and accelerate clinical skill development. Additionally, while artificial intelligence (AI) models have shown high accuracy, they still face challenges in real-world deployment, including biases, errors in unseen cases, and a lack of explainability. Our study’s findings on expert search behavior can contribute to designing AI systems that align more closely with human diagnostic strategies, thereby improving trust and usability. Future studies should address these concerns by integrating machine learning-based pattern recognition techniques to enhance diagnostic accuracy. Moreover, eye-tracking systems could provide real-time feedback to improve visual search accuracy and efficiency, distinguishing between observers who scan rapidly and those who explore slowly. Future research should also explore the impact of constructive feedback on eye movement and retinal image diagnostic accuracy in novice observers.

A key limitation of our study is the relatively small and imbalanced sample size, particularly within the expert clinician group. This constraint reflects common challenges in expertise research, where recruiting participants with specialized experience is often difficult due to limited availability and geographical constraints. While we employed robust statistical methods to mitigate the impact of group size disparities, future studies should aim to recruit larger and more diverse expert samples across multiple institutions. Expanding the participant pool will enhance generalizability and allow for more nuanced analyses of expertise-related differences in diagnostic behavior.

## 5. Conclusions

In conclusion, our study highlights differences in visual search behavior between novice and experienced clinicians and emphasizes the importance of understanding gaze patterns in medical diagnostics. These findings can inform training strategies by using expert fixation patterns to guide learners toward a more efficient visual search. Eye-tracking-based feedback and adaptive learning tools could accelerate diagnostic skill development and reduce errors through real-time, targeted support.

## Figures and Tables

**Table 1:** This table shows the mean and standard deviation (SD) of latency and peak velocity of saccades between the novice group and the experienced group during visually guided saccades.

Groups	Latency (ms)		Peak Velocity (deg/s)	
Novices	171.1 ± 33.5		308.4 ± 91.4	
	Group	Latency (ms)	Group	Peak velocity (deg/s)
	OD1	164.7 ± 29.9	OD1	305.0 ± 117.1
	OD2	169.6 ± 35.7	OD2	316.2 ± 83.2
	OD3	171.9 ± 31.4	OD3	306.8 ± 73.2
	OD4	180 ± 35.8	OD4	300.8 ± 85.7
Experienced	185.3 ± 29.3		318.6 ± 79.9	

**Table 2:** The table presents a correlation matrix between fixation and diagnostic accuracy, encompassing total dwelling time, total fixation count, and total fixation rate, comparing the novice group and the experienced group. * Shows significance after Bonferroni correction.

Groups	Total Dwelling Time	Total Fixation Count
Novices	r = 0.14; *p* = 0.42	* r = 0.54; *p* < 0.001
Experienced	r = 0.16; *p* = 0.66	r = 0.36; *p* = 0.31

**Table 3:** The table illustrates the correlation matrix between fixation and laterality accuracy, involving total dwelling time, total fixation count, and total fixation rate, for both the novice group and the experienced group. * Shows significance after Bonferroni correction.

Groups	Total Dwelling Time	Total Fixation Count	Total Fixation Rate
Novices	* r = −0.62; *p* < 0.005	r = 0.33; *p* = 0.05	r = −0.86; *p* < 0.05
Experienced	r = −0.02; *p* = 0.95	r = 0.07; *p* = 0.83	r = −0.15; *p* = 0.68

**Table 4:** Mean dwelling time with standard deviation on different areas of interest (AOIs) between experienced and novice participants during diagnostic accuracy experiment.

Area of Interest (AOI)	Experienced	Novices	*p* Value
**Bloodvessels**	1300.43 ± 1806.43	924.54 ± 1278.18	*p* < 0.006
**Disc**	2602.12 ± 1546.75	3089.86 ± 1958.48	*p* < 0.05
**Macula**	2946.17 ± 2400.12	2784.10 ± 2583.07	NS
**Other retinal regions**	3527.31 ± 2332.28	3539.80 ± 2595.04	NS

**Table 5:** Mean dwelling time with standard deviation on different areas of interest (AOIs) between experienced and novice participants during laterality accuracy experiment.

Area of Interest (AOI)	Experienced	Novices	*p* Value
**Bloodvessels**	192.33 ± 541.24	184.98 ± 426.17	NS
**Disc**	1056.37 ± 1026.85	1465.43 ± 996.42	*p* < 0.05
**Macula**	895.63 ± 916.64	626.70 ± 833.35	*p* < 0.05
**Other retinal regions**	1120.47 ± 890.30	1005.47 ± 822.57	NS

## Data Availability

The raw data supporting the conclusions of this article will be made available by the authors on request.
